# The effects of levosimendan on brain metabolism during initial recovery from global transient ischaemia/hypoxia

**DOI:** 10.1186/1471-2377-12-81

**Published:** 2012-08-24

**Authors:** Anna B Roehl, Norbert Zoremba, Markus Kipp, Johannes Schiefer, Andreas Goetzenich, Christian Bleilevens, Nikolaus Kuehn-Velten, Rene Tolba, Rolf Rossaint, Marc Hein

**Affiliations:** 1 Department of Anaesthesiology, RWTH Aachen University Hospital, Pauwelstrasse 30, Aachen, D-52074, Germany; 2 Department of Neuroanatomy, RWTH Aachen, Wendlingweg 2, Aachen, 52072, Germany; 3 Department of Neurology, RWTH Aachen University Hospital, Pauwelstrasse 30, Aachen, 52074, Germany; 4 MLHB, Medical Laboratory Bremen, Haferwende 12, Bremen, 28357, Germany; 5 Department of Experimental Animal Science, RWTH Aachen University Hospital, Pauwelstrasse 30, Aachen, 52074, Germany

**Keywords:** Levosimendan, Cerebral ischaemia, Hypoxia, Microdialysis

## Abstract

**Backround:**

Neuroprotective strategies after cardiopulmonary resuscitation are currently the focus of experimental and clinical research. Levosimendan has been proposed as a promising drug candidate because of its cardioprotective properties, improved haemodynamic effects *in vivo* and reduced traumatic brain injury *in vitro*. The effects of levosimendan on brain metabolism during and after ischaemia/hypoxia are unknown.

**Methods:**

Transient cerebral ischaemia/hypoxia was induced in 30 male Wistar rats by bilateral common carotid artery clamping for 15 min and concomitant ventilation with 6% O_2_ during general anaesthesia with urethane. After 10 min of global ischaemia/hypoxia, the rats were treated with an i.v. bolus of 24 μg kg^-1^ levosimendan followed by a continuous infusion of 0.2 μg kg^-1^ min^-1^. The changes in the energy-related metabolites lactate, the lactate/pyruvate ratio, glucose and glutamate were monitored by microdialysis. In addition, the effects on global haemodynamics, cerebral perfusion and autoregulation, oedema and expression of proinflammatory genes in the neocortex were assessed.

**Results:**

Levosimendan reduced blood pressure during initial reperfusion (72 ± 14 vs. 109 ± 2 mmHg, p = 0.03) and delayed flow maximum by 5 minutes (p = 0.002). Whereas no effects on time course of lactate, glucose, pyruvate and glutamate concentrations in the dialysate could be observed, the lactate/pyruvate ratio during initial reperfusion (144 ± 31 vs. 77 ± 8, p = 0.017) and the glutamate release during 90 minutes of reperfusion (75 ± 19 vs. 24 ± 28 μmol·L^-1^) were higher in the levosimendan group. The increased expression of *IL-6, IL-1ß TNFα and ICAM-1*, extend of cerebral edema and cerebral autoregulation was not influenced by levosimendan.

**Conclusion:**

Although levosimendan has neuroprotective actions *in vitro* and on the spinal cord *in vivo* and has been shown to cross the blood–brain barrier, the present results showed that levosimendan did not reduce the initial neuronal injury after transient ischaemia/hypoxia.

## Background

Patients under cardiac arrest undergo acute global ischaemia and acute reperfusion injury due to the return of spontaneous circulation. Although this reperfusion injury affects all organs, the heart and brain are particularly vulnerable. Neurological and cardiac complications following cardiopulmonary resuscitation (CPR) are closely associated and might aggravate cellular damage. Cerebral ischaemia activates cellular processes, including apoptosis, inflammation, inhibition of protein synthesis and increased oxidative stress, that persist despite the restoration of substrate delivery [1]. This initial neuronal injury involves disruptions in brain metabolism and the release of neurotransmitters, which activate neurotoxic cascades that can be monitored by microdialysis [2-4].

Studies have previously demonstrated that ischaemic postconditioning dramatically attenuates irreversible myocardial injury [5]. Furthermore, a reduction of the cerebral ischaemia-reperfusion injury due to ischaemic postconditioning has been previously described [6]. Interestingly, studies have estimated that every minute of lost cerebral perfusion in the human brain results in a loss of neurons that is equivalent to the loss of neurons after 3.6 years of the normal human ageing process [7]. Currently, the only valid form of therapy to improve neurological outcome is to perform therapeutic hypothermia 12 to 24 h following resuscitation [8]. Concomitant with the symptomatic therapy used to achieve return of spontaneous circulation (ROSC), therapeutic options employed to minimise the reperfusion injury have to be considered.

The ideal pharmacological intervention would be initiated during CPR and would exhibit cardiac and neuroprotective properties. Thus, a cardiac postconditioning effect may only be attained prior to the time window because the cardiac postconditioning closes two minutes after the re-establishment of spontaneous circulation [9]. The time frame for cerebral postconditioning has previously been described as a maximum of three minutes following the onset of reperfusion [10], whereas expansion of the penumbra after focal ischaemia may be reduced within the first six hours after reperfusion. Thus, sufficient effect-site concentrations of postconditioning agents should be made available at the start of the reperfusion. Levosimendan is a novel inodilator that enhances myocardial performance without resulting in substantial changes in myocardial oxygen consumption [11]. Levosimendan reduces myocardial injury if it is applied during ischaemia and early reperfusion [12]. Evidence of levosimendan’s neuroprotective properties include reduced cell death, inflammatory response and lipid peroxidation in the spinal cord and improved function after transient ischaemia [13,14]. Currently, the demonstration of a reduction of primary and secondary injury after brain trauma has been limited to *in vitro* models [15]. The protective effects of levosimendan are mediated in the heart by activation of the PI3K pathway, the inducible nitric oxide synthase and mitochondrial ATP-dependent potassium channels (mK_ATP_). This also results in a clearly vasodilation [16-18]. The important role of mK_ATP_ channels in cerebral ischaemia-reperfusion injury and positive action of other activators (diazoxide) on neuronal injury, spark hopes of neuroprotective effects of levosimendan [19,20].

Based on the promising results of levosimendan, the aim of the present study was to test the hypothesis that levosimendan reduces initial ischaemic/hypoxic neuronal injury in the neocortex by postconditioning. We employed a model of bilateral carotid occlusion with an additional reduction of inspired oxygen concentration. Changes in the metabolite and substrate levels, haemodynamics, regional perfusion, blood–brain barrier dysfunction and local inflammatory response were measured by microdialysis, blood pressure measurements, laser Doppler flow, and claudin 5/tight junction protein and interleukin expression, respectively.

## Methods

### Instrumentation

All of the experiments were performed in accordance with the German legislation governing animal studies and followed the Guide for the Care and Use of Laboratory Animals [21]. Official permission for these studies was granted from the governmental animal care and use office (Landesamt für Natur-, Umwelt- und Verbraucherschutz Nordrhein-Westfalen, Recklinghausen, Germany, Protocol No. 8.87-50.10.55.09.064).

A previously established protocol was modified to investigate the effects of levosimendan on ischaemic brain injury [22]. Thirty male Wistar rats (Charles River, Sulzfeld, Germany, 300–350 g) were anaesthetised by an intraperitoneal injection of 1.5 g/kg urethane (Sigma-Aldrich Chemie GmbH, Steinheim, Germany). After a tracheotomy, the animals were ventilated with 30% oxygen under the pressure-controlled mode (P_max_ 13 mmHg, PEEP 5 mm Hg) using a commercially available respirator (Evita 2, Draeger, Luebeck, Germany) with a respiratory frequency between 40 and 55 min^-1^. The right femoral vein was catheterised with a 22 G cannula (Leader Flex, Vygon, Germany) using Seldinger’s technique for continuous drug administration and blood withdrawal. The mean arterial pressure (MAP) was measured with a 1.4 F pressure catheter (SPR-671, Millar Instruments, Houston, Texas, USA) that was placed through the right femoral artery. The heart rate (HR) was calculated from the ECG signal. All of the data were recorded using an acquisition and analysis system (Power Lab 8/30, LabChart 6 Pro v 6.11; ADInstruments, Colorado Springs, USA). A continuous infusion of 4 ml kg^-1^ h^-1^ Ringers solution was administered to compensate for the perioperative fluid loss. To maintain body temperature, the animals were placed on a back-coupled heating pad (MLT1403 and TCAT-2 Controller, ML 295/R, Physitemp Instruments, USA). Both of the common carotid arteries were identified in the supine position and isolated from the attached vagal nerve. To induce carotid clamping, a 2–0 silk suture (Fine Science Tools, Heidelberg, Germany) was placed around each common carotid artery.

**Figure 1:**
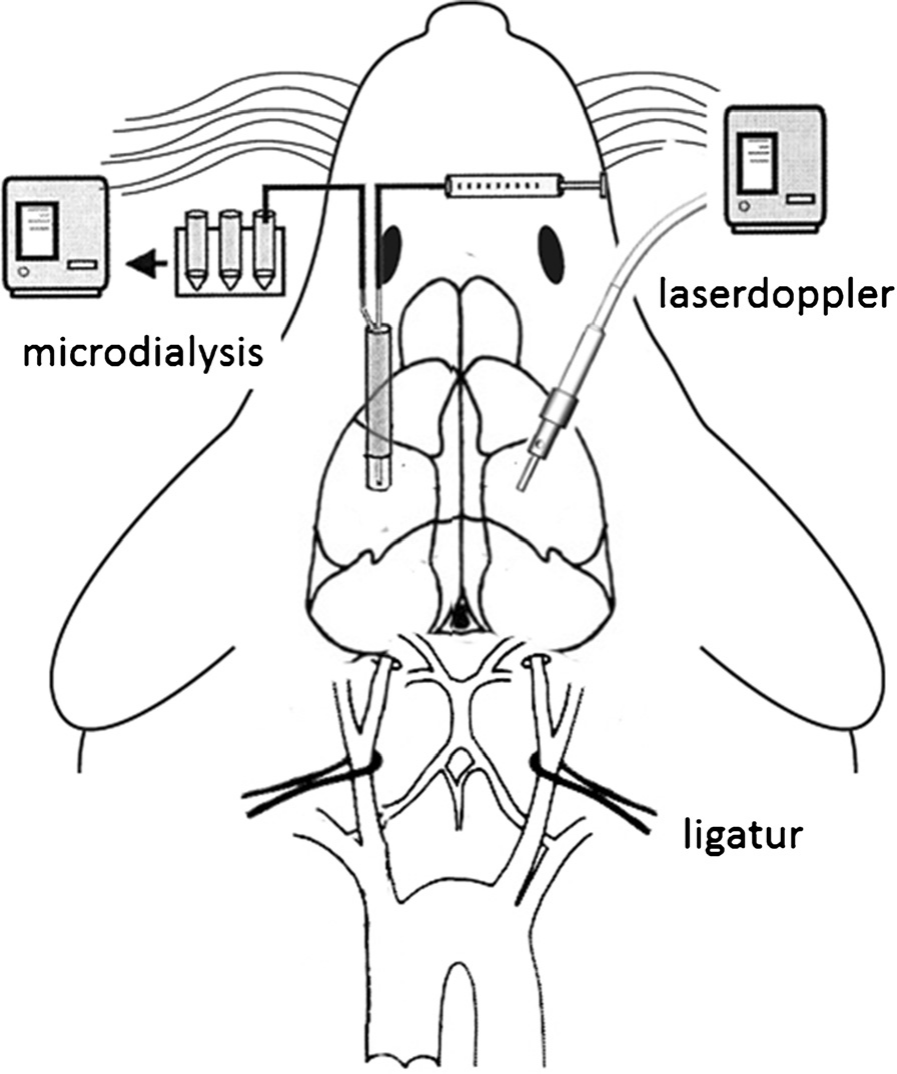
Schematic of the experimental setting.

In the prone position, the heads of the rats were fixed in a stereotactic apparatus (Figure 1). A laser Doppler probe (moor VMS-LDF1, Moor Instruments Inc., Devon, Great Britain) was positioned 5.5 mm lateral-right and 1 mm caudal from the bregma. The cranium was thinned using a small hand drill (Dremel 300 Series, Dremel, Leinfeld-Echterdingen, Germany), and the laser Doppler probe was positioned in a vertical position and fixed with a commercially available instant adhesive (UHU, Bühl, Germany) and activator spray (JAMARA Modeltechnik, Aichstetten, Germany). The recorded perfusion units (PFU) were normalised (100%) to values at baseline that were recorded 10 min prior to the induction of ischaemia/hypoxia. The impairment of cerebral autoregulation was characterised by the autoregulatory index (ARI), which was calculated from the slope obtained from the linear regression analysis of the relative change of PFU (%) and MAP (%) during reperfusion [23]. The cerebral vascular resistance (CVR) was calculated as the ratio of the MAP to the normalised PFU.

The left somatosensory cortex was partially exposed by a burr hole located 2–3 mm caudal from the bregma and 3–4 mm lateral from the midline (Figure 1). The dura was partially opened with a needle to place a microdialysis probe with a membrane length of 2 mm, an outer diameter of 0.5 mm and a cut–off at 20000 DA (CMA 12, 2 mm membrane length, CMA Microdialysis, Solna, Sweden). The microdialysis method has been previously described in detail by Ungerstedt et al. [24]. The microdialysis catheter was continuously perfused with a dialysate containing 147 mmol/l NaCl, 2.7 mmol/l KCl, 1.2 mmol/l CaCl_2_ and 0.85 mmol/l MgCl_2_ (Perfusion fluid CNS, CMA Microdialysis) at a flowrate of 2 μl*min^-1^ using a precision infusion pump (CMA 102, CMA Microdialysis). The samples were collected in 10 min intervals and frozen at -20°C until the analysis. The thawed and centrifuged dialysate samples were analysed enzymatically with a chemistry analyser (CMA 600 Microdialysis Analyser, CMA Microdialysis, Schweden) for lactate, pyruvate, glucose and glutamate concentrations. Prior to the experiments and at the end of the experiments, the relative recovery rates for each substance were determined with a calibration solution (Calibrator, CMA Microdialysis) and applied to the experimental values. For glutamate, the area under the concentration curve was calculated to quantify the relationship between release and uptake.

### Experimental protocol

Due to the transient increases in metabolite concentrations from placing the microdialysis probe in the brain, an equilibration period of 60 min was required. Three baseline measurements of haemodynamic data and corresponding cerebral microdialysates were each sampled within a 10 min interval. To prevent the rats from spontaneous breathing triggered by hypoxia, 2 mg kg^-1^ of rocuronium (Esmeron, Schering-Plough, Kenilworth, NJ, USA) was injected i.v. The rats were randomly divided into three groups using an envelope system. After 10 min of ischaemia, the first group received a bolus of 24 μg kg^-1^ levosimendan (Simdax® 2.5 mg/ml, Orion Pharma, Espoo, Finland), which was administered over a period of 20 min, followed by a continuous infusion of 0.2 μg kg^-1^ min^-1^ throughout the experiment. The control group received an equivalent amount of 0.9% NaCl. The sham rats were not subjected to cerebral ischaemia/hypoxia but did receive an equivalent amount of NaCl as the control group. To induce ischaemia and hypoxia, both ligatures around the carotid arteries were closed, and the inspired oxygen concentration was reduced to 6%. Although the haemodynamic variables were recorded during ischaemia/hypoxia within a 5 min interval, the dialysate was collected over the entire 15 min of hypoxia/ischaemia. After 15 min, the ligatures were released, and the inspired oxygen concentration values normalised (30%). During the following 90 min of reperfusion, the data were recorded every 10 min, and the dialysate was also collected during these intervals.

**Figure 2:**
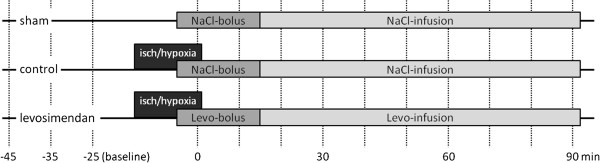
**A study design diagram: the treatment of the groups related to the procedures.** The vertical lines mark the measurement time points.

At the end of the measurement period, the abdomen and thoracic cavity were opened while the rat was in the supine position. The left ventricle of the beating heart was cannulated (22 G, Microlance, Becton Dickinson, Espana), and 4 ml of blood was collected for further analysis. The animals were transcardially perfused with 50 ml of cold (4°C) Ringers solution. The brain was excised and cut into seven 2 mm cross-sectional slices. Two slices were used to calculate the percentage of water content, and 2 slices (the fourth and fifth) were immediately snap-frozen in liquid nitrogen and stored at −80°C for real-time PCR examination. The rest of the slices were used for further tissue analysis of the levosimendan concentration (Figure 2).

### Levosimendan brain tissue concentration

The brain tissue samples were homogenised in 1 ml of TRIS buffer (Tris-Base 20 mmol/l mM, NaCl 150 mM, EDTA 1 mM) per gram of wet weight. The homogenates (500 μl per 50 mg of tissue) were extracted at a neutral pH with ethyl acetate. After evaporation and resuspension in the mobile phase (acetonitrile/1% acetic acid), separation of levosimendan was achieved using a reversed-phase C18 column (the retention time was 1.4 min) with zonisamide as the internal standard. Specific ions were detected with an API4000 tandem MS/MS in the negative multiple reaction mode (Sciex/Applied Biosystems, Foster City, CA, USA).

### S100ß protein analysis

S100ß serum levels at the end of the reperfusion were analysed by an enzyme-linked immunosorbent assay (ELISA) using a commercially available kit. The assays were performed according to the manufacturer’s instructions (YK150, SCETI, Tokyo, Japan).

### Messenger RNA quantification by real-time RT-PCR

Gene expression levels of the pro-inflammatory cytokines *tumour necrosis factor α (TNFα)*, *interleukin 6 (IL-6), interleukin 1ß (IL-1β)* and *intercellular adhesion molecule 1 (ICAM-1)* in the cortex close to the laser Doppler position were determined by quantitative real-time polymerase chain reaction (qRT-PCR). Disruption of the blood–brain barrier was assessed by the gene expression of *claudin 5 (Cldn-5)* and *tight junction protein 1 (Tjp-1).* Total RNA was extracted using a commercially available RNA/Protein extraction kit (NucleoSpin® RNA/Protein, Machery-Nagel, Düren, Germany) and reverse-transcribed into cDNA using a high-capacity reverse transcription kit (Applied Biosystems®, Carlsbad, CA, USA). The PCR reaction was performed using 50 ng of cDNA (TaqMan® universal PCR mix, Applied Biosystems®) and specific TaqMan® probes for *IL-1β* (Rn00580432_m1), *IL-6* (Rn01410330_m1), *TNF-α* (Rn00562055_m1), *ICAM-1* (Rn00564227_m1), *Cldn-5* (Rn01753146_s1), *Tjp-1* (Rn02116071_s1) and the housekeeping gene *hypoxanthin-guanin-phosphoribosyltransferase* (*HPRT*, Rn01527840_m1) on a StepOne-Plus® Cycler (Applied Biosystems®). The relative quantity (RQ) values were calculated according to the ΔΔCt method, which reflects the differences in the threshold for each target gene relative to *HPRT* and the sham-operated rat.

### Statistical analysis

We used a multivariate analysis for repeated measurements and a univariate analysis using Scheffe’s or Kruskal-Wallis post hoc test for pairwise comparisons between the groups (depending on the results obtained from Levene’s test for the equality of variances). A one-sample *t* test was used to compare the RQ values with the sham-operated rats (RQ = 1). The effects of ischaemia/hypoxia and levosimendan on cerebral autoregulation were determined using multiple linear regression analysis (SPSS 19; IBM Corporation, Somers, NY, USA). The RQ values were plotted as box and whisker graphs displaying the 5th and 95th percentiles, whereas all of the other results were presented as the mean and SEM (Prism 5.01, GraphPad Software, San Diego, CA). A p-value of <0.05 was considered to be statistically significant.

## Results

Six of the 30 rats died during the experimental procedure because of several factors, including procedure-induced injury of the carotid artery, cardiac failure, ventricular fibrillation and brain death. Nine rats were treated with levosimendan, 9 rats received NaCl (control group) and sham-operations were performed on 6 rats. Levosimendan crossed the blood–brain barrier and reached a tissue concentration of 0.17 ± 0.13 ng g^-1^.

### The effects of ischaemia/hypoxia

**Figure 3:**
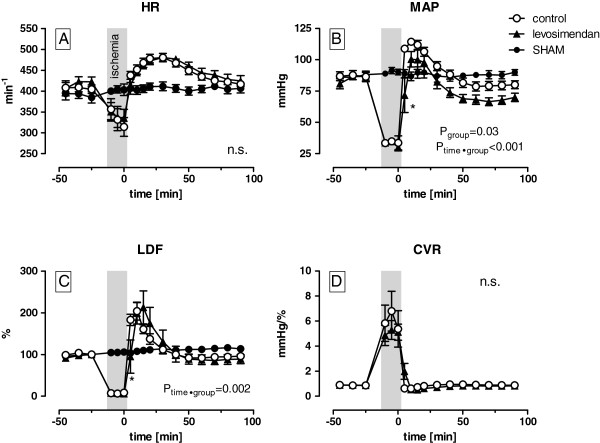
The effects of levosimendan on the haemodynamics and cerebral perfusion during 15 min of bilateral cerebral ischaemia/hypoxia and 90 min of reperfusion (the p-values indicate differences between the control and levosimendan treatment group as assessed.

**Figure 4:**
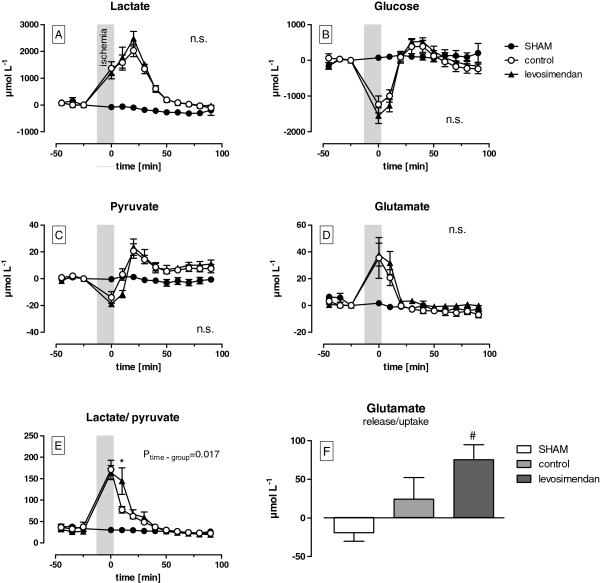
**The effects of levosimendan on the extracellular concentrations of lactate [A], glucose [B], pyruvate [C], glutamate [D] and the lactate/pyruvate ratio [E] during 15 min of bilateral cerebral ischaemia/hypoxia and 90 min of reperfusion.** The amount of glutamate release and uptake was quantified by the area under the curve from plot D (The p-values indicate differences between the control and levosimendan from an ANOVA; * p < 0.05 vs. control, # p < 0.05 vs. sham).

During ischaemia/hypoxia, the HR decreased from 407 ± 1 to 325 ± 10 min^-1^ and MAP from 84 ± 3 to 32 ± 2 mmHg with no differences between the levosimendan and the control group. The LDF signal had dropped to 6-8% of the baseline values in both of the groups. No effect of the levosimendan bolus during this period was evident (Figure 3). Both, the levosimendan as well as the control group showed a relevant increase in lactate (+1282 ± 86 μmol L^-1^) and glutamate (+37 ± 1 μmol L^-1^) levels, whereas the levels of pyruvate (−16 ± 2 μmol L^-1^) and glucose (−1391 ± 158 μmol L^-1^) were markedly decreased. Thus, the lactate/pyruvate ratio increased by 5 times during ischaemia/hypoxia (Figure 4). No differences were found between the groups.

### The effects of reperfusion

**Figure 5:**
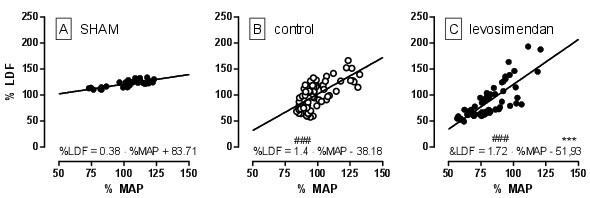
**A correlation of the relative change of the laser Doppler flow (%LDF) and the mean arterial pressure (%MAP) during reperfusion.** The slope of the linear regression reflects the autoregulatory index (^###^ p < 0.001 vs. sham; ***P < 0.001 vs. control for the intercept). by an ANOVA and post hoc test at various time points, * p < 0.05). HR – heart rate, MAP – mean arterial pressure, LDF – laser Doppler flow, CVR – cerebral vascular resistance.

During early reperfusion, an overshoot in the HR, MAP and LDF may be detected with peak values after 10–25 min (Figure 3). The levosimendan group showed a significant delayed (5 min) and smaller increase in the MAP compared with the control group (72 ± 14 vs. 109 ± 2 mmHg, p = 0.03). In addition, the LDF increase to the maximum values was delayed by 5 min in the levosimendan group (p = 0.002) but reached levels that were comparable with the control group. Moreover, the mean arterial pressure of the levosimendan-treated rats remained 12 ± 2 mmHg (p = 0.03) lower throughout the reperfusion period, whereas no differences in the LDF amplitudes could be measured between the groups. Ischaemia/hypoxia in both groups resulted in an impairment in the cerebral autoregulation, which was demonstrated by a higher ARI in the control (1.4) and levosimendan (1.72) group compared with the sham group (0.38, p < 0.001). There were no differences between the treatment groups (Figure 5). Although the elevation of the regression line between the LDF and the MAP was significantly different between the groups, no effect of levosimendan on the CVR was observed (Figure 4D). The HR normalised after 90 min and there were no differences between the groups.

Reperfusion resulted in a further increase in the concentration of lactate and pyruvate in the dialysate within the first 20 min, but this increase was followed by a continuous decrease (Figure 4A and C). In addition, the lactate/pyruvate ratio decreased less during early reperfusion following levosimendan treatment compared with the control group (144 ± 31 vs. 77 ± 8, p = 0.017, Figure 4E). Moreover, the glutamate returned to baseline values within 20 min of reperfusion, whereas glucose concentrations returned to baseline values within 50 min of reperfusion. Similar to the pyruvate levels, the glucose levels transiently increased within the first 20 min. Furthermore, the levosimendan-treated animals displayed more glutamate release (75 ± 19 μmol·L^-^1) compared with the control (24 ± 28 μmol·L^-1^, p = 0.29) and sham (-19 ± 11 μmol·L^-1^, p = 0.02, Figure 4D).

**Figure 6:**
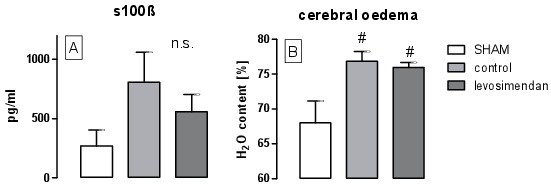
Serum levels of s100ß after 90 min of reperfusion (A) and the extent of cerebral oedema characterised by the water content (B) (# p < 0.05 vs. sham).

**Figure 7:**
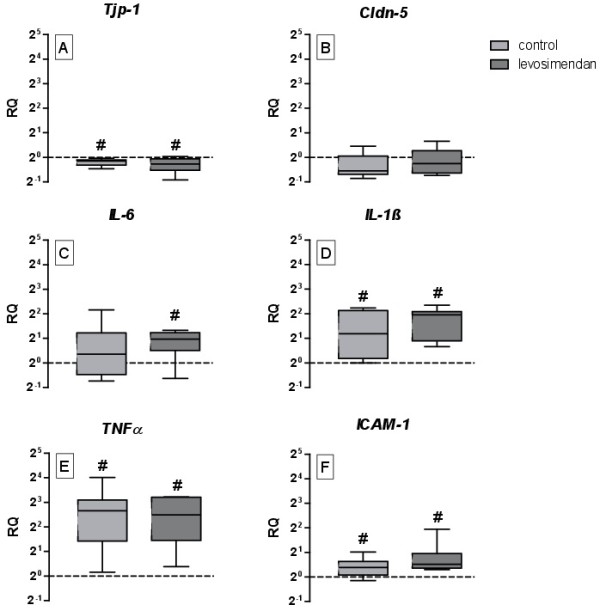
**The effects of levosimendan on the gene expression of the *tight junction protein 1*(A), *claudin 5 *(B), *interleukin 6 *(C), *interleukin 1ß *(D), *tumour necrosis factor α *(E) and *intercellular adhesion molecule 1 *(F) in the cortex at the end of the reperfusion period (# p < 0.05 vs. sham).**

Despite the higher levels of s100ß after ischaemia/hypoxia, no significant differences between the groups could be detected after 90 min of reperfusion (Figure 6A). Although the water content increased significantly from 68 ± 7% in the sham group to 76 ± 3% after ischaemia/hypoxia (p = 0.007), there were no differences between the levosimendan-treated group and the control group (Figure 6B). Interestingly, cerebral oedema may be a consequence of reduced expression of *Tjp-1* (RQ = 0.84 ± 0.03, p = 0.01, Figure 7A). In addition, we observed reduced expression of *Cldn-5 RQ*, but the results were not significant (RQ = 0.88 ± 0.07, p = 0.09, Figure 7B). Ischaemia/hypoxia also led to an increased inflammatory response in the cortex after 90 min of reperfusion. Indeed, we observed pronounced upregulation of *TNFα* and *IL-1ß* by factor 62 (p = 0.01) respectively 8 (p = 0.02) and a moderate threefold upregulation of *ICAM-1* (p = 0.03) in both of the groups, whereas *IL-6* levels were only elevated by factor 3.5 in the levosimendan-treated group (p = 0.02). Beside this, levosimendan did not cause any further significant effects on gene expression compared with the control group (Figure 7).

## Discussion

The aim of the present study was to evaluate the effects of levosimendan on brain metabolism, perfusion and inflammatory response resulting from a defined hypoxia/ischaemia injury and to characterise the time-course during subsequent re-oxygenation. We found that levosimendan did not reduce the initial ischaemic/hypoxic neuronal injury if it was administered after the insult and during resuscitation.

Ischaemia/hypoxia leads to a primary energy failure that is accompanied by the dysfunction of ATP-dependent ion channels (Na^+^/Ca^2+^, Ca^2+^/H^+^) and an increased intracellular Ca^2+^ concentration [25,26]. Subsequent glutamate release activates postsynaptic AMPA and NMDA receptors, which induce intracellular sodium and calcium overflow that may be detected prior to anoxic depolarisation [27]. As a consequence of all this cellular oedema and the activation of different proteases, lipases, endonucleases and the generation of free radicals is induced. Importantly, the restoration of blood flow and oxygenation will restore oxidative metabolism within 30 to 60 min, which suggests a therapeutic window during which the neurotoxic cascade can be inhibited. Most neurons will die as a consequence of secondary energy failure, which occurs 6 to 15 h after injury [4]. The magnitude of primary cell death is dependent on the severity and duration of ischaemia/hypoxia and could not be observed in the neocortex within the first 13 min [28]. In the present study, we found that an insignificant increase of s100ß could be observed after 90 min of reperfusion. Thus, the neuroprotective effects of levosimendan observed in the neocortex within the first few hours could only be evaluated based on the monitoring of triggers of cell death (i.e., glutamate release, energy metabolism and inflammation).

Previous studies have demonstrated that it is possible to reduce glutamate release during ischaemia/hypoxia by treatment with tiagabine [29], dantrolene [30], nimodipine [31] or magnesium [32]. In principle, the activation of mK_ATP_ channels should result in smaller increases in intracellular Ca^2+^ levels and glutamate release during ischaemia. Thus, levosimendan may be as effective as diazoxide [33,34]. Studies have suggested that mK_ATP_ agonists may be beneficial for the treatment of brain disorders that are associated with low ATP levels [35], and levosimendan has been shown to be neuroprotective in the spinal cord when applied prior to [13] or during ischaemia [14]. Preservation of the energy status displayed an important mechanism of protection, but was independent of vasodilatation [36].

The failure of levosimendan to affect neuroprotection the parameters determined during the present study may be related to the low intracerebral concentrations achieved in the current protocol. Although efficient serum concentrations for cardiac effects (32 μg/L) were achieved, the tissue concentration in the brain reached only 12% of the concentration in the heart. Although this concentration (0.17 ng g^-1^) was six times higher compared with animals with an intact blood–brain barrier [37], it was not sufficient in the *in vitro* model to reduce the primary or secondary injury after trauma. In the traumatic brain model injury model, a 100-fold higher concentration was required to achieve significant affects [15]. The low cerebral concentrations could be a consequence of the rapid redistribution of the levosimendan bolus [37] and the delayed disruption of the blood–brain barrier related to the insult [22,38], which appears to be a prerequisite to achieve higher levosimendan levels within the brain.

Other reasons for the ineffectiveness of levosimendan might be related to the unique differences of individual animal models, such as the observation period after the insult, the methods used to describe the neuronal injury and the region of interest. For example, the basal ganglia and the hippocampus are more susceptible to ischaemia [28]. The proposed protective effects of levosimendan might become visible after a longer observation period and may not be associated with reduced glutamate release [39]. The magnitude of neuronal injury after 15 min of ischaemia/hypoxia should be questioned because no lasting effect on metabolism, glutamate release or s100ß increase was observed. Indeed, only the increase in inflammation and cerebral oedema and the disruption of auto regulation and the blood–brain barrier account for cerebral injury. However, if the ischaemia/hypoxia is prolonged in this model, animals may die from cardiovascular complications or brain death. Thus, modifications to the current protocol for levosimendan administration and a longer observation period are necessary for further validation.

The neuroprotective effects of different drugs might be related to effects on the vasculature. For example, the protective effects of nimodipine and magnesium are associated with decreased cerebral blood flow during reperfusion. Studies have previously shown that controlled reperfusion alone can reduce neuronal injury [40]; thus, the protective actions of drugs may be mediated by a similar mechanism. Indeed, levosimendan affects cerebral perfusion pressure and flow [41]. Although levosimendan delayed reperfusion, we observed an upward shift in the relationship between CBF and MAP, which might counterbalance levosimendan’s direct protective actions. In this context the activation of mK_ATP_ channels and increase of NO release in the vessels by levosimendan was effective and led to vasodilation in the brain as described earlier in an animal model of subarachnoid haemorrhage [42]. In addition, the delayed reperfusion during levosimendan treatment may explain the slower normalisation of the lactate/pyruvate ratio. The relevance of this difference is questionable, however, because no differences in the extracellular glucose concentrations were observed.

Other properties associated with cerebral injury were unaffected by levosimendan treatment. The lack of a disturbance of cerebral auto regulation and disruption of the blood–brain barrier resulted in similar cerebral oedema responses between the groups. Similarly, diazoxide also demonstrated protective effects in this context [43], where activation of K_ATP_ channels reduced the permeability of the BBB and down regulation of occludin after hypoxia [44]. These differences might be explained by a diverse affinity to receptor subtypes of the substances, which have not been investigated in detail.

Although levosimendan demonstrated anti-inflammatory actions in sepsis, myocardial reperfusion injury and ARDS, no effects on the expression of inflammatory genes in the neocortex were observed. Because inflammation aggravates neuronal injury [45], the protective effects of levosimendan under inflammatory conditions are more unlikely.

## Conclusion

In conclusion, levosimendan did not exhibit neuroprotective actions in the initial phase after experimental ischaemic/hypoxic cerebral injury. We did not find any relevant effects on metabolism, release of glutamate, inflammation, auto regulation or the integrity of the blood–brain barrier. Moreover, no aggravation of brain injury was found.

## Competing interests

The authors declare that they have no competing interest.

## Authors’ contributions

AR, NZ and MH conceived of the study, participated in the study’s design and coordination, performed the statistical analysis and drafted the manuscript. AR, NZ, CB and MH conducted the experimental laboratory work. RR and RT helped to draft the manuscript. MK, JS and AG participated in the study’s design and helped to draft the manuscript. WKV established new laboratory measurements for levosimendan detection and helped to draft the manuscript. All authors read and approved the final manuscript.

## Pre-publication history

The pre-publication history for this paper can be accessed here:

http://www.biomedcentral.com/1471-2377/12/81/prepub
